# Complete Genome Sequence of Tick-Borne Encephalitis Virus Strain A104 Isolated from a Yellow-Necked Mouse (*Apodemus flavicollis*) in Austria

**DOI:** 10.1128/genomeA.00564-13

**Published:** 2013-08-08

**Authors:** Stefan Frey, Sandra Essbauer, Gudrun Zöller, Boris Klempa, Manfred Weidmann, Gerhard Dobler, Martin Pfeffer

**Affiliations:** Bundeswehr Institute of Microbiology, Munich, Germanya; Institute of Virology, Slovak Academy of Science, Bratislava, Slovakiab; Institute of Medical Virology, Helmut-Ruska-Haus, Charité University Hospital, Berlin, Germanyc; Department of Virology, University Medical Center Göttingen, Göttingen, Germanyd; Institute of Animal Hygiene and Veterinary Public Health, University of Leipzig, Leipzig, Germanye

## Abstract

Tick-borne encephalitis virus (TBEV) strain A104 was isolated from the brain of a yellow-necked mouse in Austria in 1990. The complete genome sequence was 11,097 nucleotides long. Comparison with TBEV prototype strain Neudoerfl showed 32 amino acid exchanges and the absence of an internal poly(A) stretch within the 3′ noncoding region.

## GENOME ANNOUNCEMENT

Tick-borne encephalitis virus (TBEV) is a human-pathogenic flavivirus within the family *Flaviviridae* (1). The genome of TBEV consists of a single-stranded 11-kb (+) RNA coding for one large polyprotein, yielding three structural proteins (C, prM, and E) and seven nonstructural proteins (NS1, NS2a, NS2b, NS3, NS4a, NS4b, and NS5) (2). Three subtypes of TBEV with different phylogenetic and pathogenic characteristics have been distinguished, the Western subtype (W-TBEV), the Siberian subtype (S-TBEV), and the Far-Eastern subtype (FE-TBEV) (3). In the past decade Europe has suffered an increase of tick-borne encephalitis (TBE), which can cause a disease of the central nervous system (4). In the unvaccinated population of Austria, a total of 8,493 cases of TBE have been reported from 1972 to 2011, with an average incidence rate of about 6 cases per 100,000 population (5). Meanwhile, vaccination has reached >85%, thereby dropping the Austrian incidence rate to 0.9 per 100,000 population (5). Here we describe a new complete genome sequence of a TBEV strain from Austria, which is the first isolate since strain Neudoerfl (1970; accession number U27495) and also the first TBEV strain isolated from a free-ranging mouse for which the complete sequence was determined.

The TBEV strain A104 was isolated from the brain of a yellow-necked mouse (*Apodemus flavicollis*) trapped in Wagnitz (Austria) in 1990 (6). The virus was passaged two times in mouse brains. After one passage in VeroB4 cells, nucleic acid was extracted from the infected cell culture supernatant. Full-genome sequencing was performed using protocols described elsewhere (S. Frey, S. Essbauer, G. Zöller, B. Klempa, M. Weidmann, G. Dobler, and M. Pfeffer, submitted for publication). Nucleotide sequences were determined by using an ABI PRISM 3130 genetic analyzer.

Strain A104 had 97.6% nucleotide identity to the Western TBEV prototype strain Neudoerfl. The most remarkable nucleotide heterogeneity was shown in the 3′ noncoding region (NCR). At position 10485 of strain A104, a poly(A) tail of A_3_-C-A_6_ was observed, which is much shorter than that from strain Neudoerfl, A_3_-C-A_48_ (7). The origin of heterogeneity in the 3′ NCR was considered to be associated with virus propagation *in vitro* as well as polymerase stumbling across extensive secondary structures of the viral RNA (8, 9). The deduced polyprotein of strain A104, with a length of 3,414 amino acids (aa), differed by 32 aa from the polyprotein of strain Neudoerfl. Positions with aa changes were not equally scattered along the viral genome. NS2a (4 aa exchanges), NS3 (8 aa exchanges), and NS5 (9 aa exchanges) showed the highest relative aa change rates, whereas preM and NS4a were 100% identical. Phylogenetic analysis of A104 with other available complete TBEV genomes from the W-TBEV showed that the Austrian strain grouped together with strain AS33 (GQ266392) (10) from southeastern Germany and three TBEV strains from Slovakia (KC835595 to KC835597) (Frey et al., submitted). Interestingly, strain A104 showed a closer phylogenetic relationship with the German strain AS33 than with the geographically closer Austrian strain Neudoerfl. The latter finding is concordant with data from neighboring Slovenia, Slovakia, and Germany showing that geographically close does not necessarily mean phylogenetically close (11, 12; Frey et al., submitted). Although this would be expected in a rodent-associated tick-borne virus, other factors will likewise drive geographical distribution of TBEV (11, 12).

### Nucleotide sequence accession number.

The complete genome sequence of TBEV strain A104 has been deposited in GenBank under the accession number KF151173.
